# Prediction of the Bending Strength of a Composite Steel Beam–Slab Member Filled with Recycled Concrete

**DOI:** 10.3390/ma16072748

**Published:** 2023-03-29

**Authors:** Mohammed Chyad Liejy, Ahmed W. Al Zand, Azrul A. Mutalib, Ali A. Abdulhameed, A. B. M. A. Kaish, Wadhah M. Tawfeeq, Shahrizan Baharom, Alyaa A. Al-Attar, Ammar N. Hanoon, Zaher Mundher Yaseen

**Affiliations:** 1 Department of Civil Engineering, Universiti Kebangsaan Malaysia (UKM), Bangi 43600, Malaysia; 2 Energy Research Unit, Al-Hawija Technical Institute, Northern Technical University, Kirkuk 36001, Iraq; 3 Department of Reconstruction and Projects, University of Baghdad, Baghdad 10071, Iraq; 4 Faculty of Engineering, Sohar University, Sohar 311, Oman; 5 Northern Technical University, Mosul 41002, Iraq; 6 Civil and Environmental Engineering Department, King Fahd University of Petroleum & Minerals, Dhahran 31261, Saudi Arabia

**Keywords:** composite system, cold-formed section, recycled aggregates, flexural strength, beam–slab system, PDS slab

## Abstract

This study investigated the structural behavior of a beam–slab member fabricated using a steel C-Purlins beam carrying a profile steel sheet slab covered by a dry board sheet filled with recycled aggregate concrete, called a CBPDS member. This concept was developed to reduce the cost and self-weight of the composite beam–slab system; it replaces the hot-rolled steel I-beam with a steel C-Purlins section, which is easier to fabricate and weighs less. For this purpose, six full-scale CBPDS specimens were tested under four-point static bending. This study investigated the effect of using double C-Purlins beams face-to-face as connected or separated sections and the effect of using concrete material that contains different recycled aggregates to replace raw aggregates. Test results confirmed that using double C-Purlins beams with a face-to-face configuration achieved better concrete confinement behavior than a separate configuration did; specifically, a higher bending capacity and ductility index by about +10.7% and +15.7%, respectively. Generally, the overall bending behavior of the tested specimens was not significantly affected when the infill concrete’s raw aggregates were replaced with 50% and 100% recycled aggregates; however, their bending capacities were reduced, at −8.0% and −11.6%, respectively, compared to the control specimen (0% recycled aggregates). Furthermore, a new theoretical model developed during this study to predict the nominal bending strength of the suggested CBPDS member showed acceptable mean value (0.970) and standard deviation (3.6%) compared with the corresponding test results.

## 1. Introduction

The building industry is continually developing new composite structural members to replace conventional members (reinforced concrete, steel, and wood timber) for a variety of reasons, including cost, availability of materials in the market, ease of fabrication, higher strength, better durability, and environment effects [1]. In 1989, Wright et al. [2] studied the composite structural performance of a profiled steel sheet deck slab system with cementitious dry board sheet, usually known as a “PSSDB or PDS system”. Later, several researchers tested the PSSDB system adopted in the floors, walls, and roofs of residential and commercial buildings [3,4,5,6]. The influence of filling a one-way PSSDB slab with geopolymer and normal concrete was experimentally investigated [7,8]. In addition, Al-Shaikhli et al. [9,10] studied the bending behavior of concrete-filled PSSDB slabs using the two-way slab concept. However, PSSDB deck slabs (with/without infill concrete) are usually provided with a small depth cross-section that subjects them to a high-deflection failure scenario under a regular static load, which means they can be only used over a limited spanning length. Therefore, to carry a heavier load and/or adapt to a long span, concrete-filled PSSDB slabs must be supported by a beam, similar to the I-steel beam–slab concept [11,12,13,14]

Furthermore, concrete-filled steel tube (CFST) members showed sufficient structural behavior under different types of bending loads [15,16,17,18]. Using slender steel tube sections in composite CFST members will reduce the structural member’s cost, but it will buckle at an early loading stage compared to the non-compact and compact tubes’ sections. Thus, the slender steel tube’s section in the CFST system usually needs to be strengthened using additional steel stiffeners to delay and/or prevent buckling failure [19]; this strengthening process increases the cost. Therefore, recent research adopted cold-formed steel C-Purlins in the CFST beam’s system, as the lips of this section act as internal stiffeners [20,21]. Generally, researchers have adopted different waste materials as recycled aggregates to replace raw aggregates in CFST columns’ concrete [22,23,24,25] beams [26,27,28], which had similar performances to those filled with normal concrete, but slightly less ultimate strength. Such waste materials include crushed concrete aggregate (CCA) [29,30,31], crumbed rubber aggregate (CRA) [32,33,34], and fine glass aggregate (FGA) [35,36,37,38,39,40,41]. Moreover, the expanded polystyrene (EPS) beads used in the concrete mixtures partially replaced the raw aggregates, which reduced the self-weight of structural elements [39,42,43,44].

According to the literature, existing studies investigated the bending behavior of CFST beams and concrete-filled PDS slabs separately. Recently, Liejy et al. [45] investigated the effects of combining these two structural members in a single composite beam-slab system (CBPDS system), where they studied the influence of using normal concrete as infill material with no additional analytical and theoretical investigation for this type of composite system [45]. Therefore, the two main objectives of this study are: First, to investigate the bending behavior of CBPDS specimens filled with the concrete materials that contain high percentages of lightweight recycled aggregates to reduce the specimen’s overall cost, self-weight, and environment improvement (by recycling more waste material in the construction field). Specifically, using combinations of different types of recycled aggregates (CCA, CRA, FGA, and EPS) replaced 50% and 100% of the infill concrete material’s raw fine and coarse aggregates, respectively. Second, to develop a new theoretical model to predict the nominal bending strength of this type of composite beam–slab system (CBPDS specimens), for practical use by engineers who adopt this system in the future. Furthermore, as this study is limited to an experimental approach, the effects of more parameters need to be studied in the future, including using numerical approaches (finite element models) [21,46].

## 2. Experimental Work

### 2.1. Specimens Preparation

**Figure 1:**
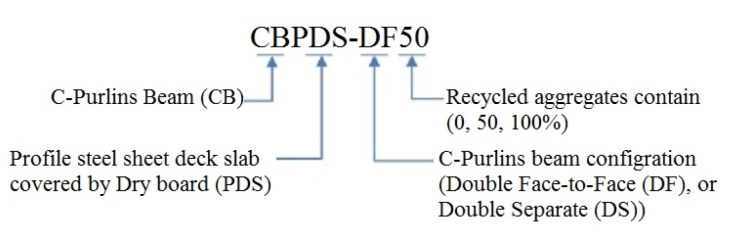
Nomenclature of specimen.

**Figure 2:**
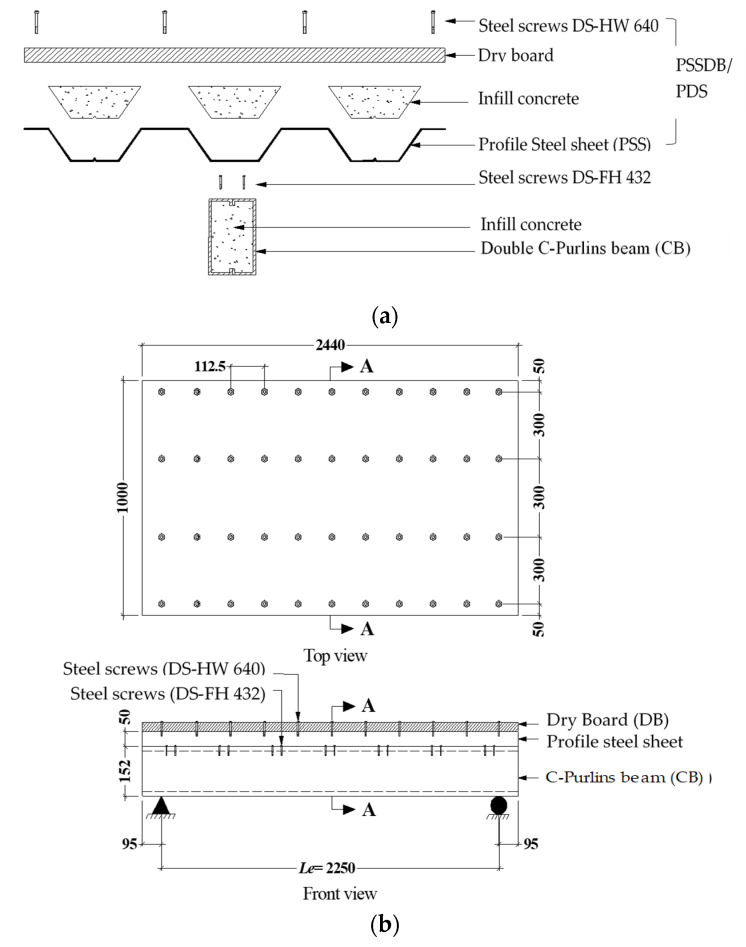
The composite CBPDS system (units in mm). (**a**) Components of CBPDS specimen; (**b**) Combination of the beam (CB) and slab (PDS).

**Figure 3:**
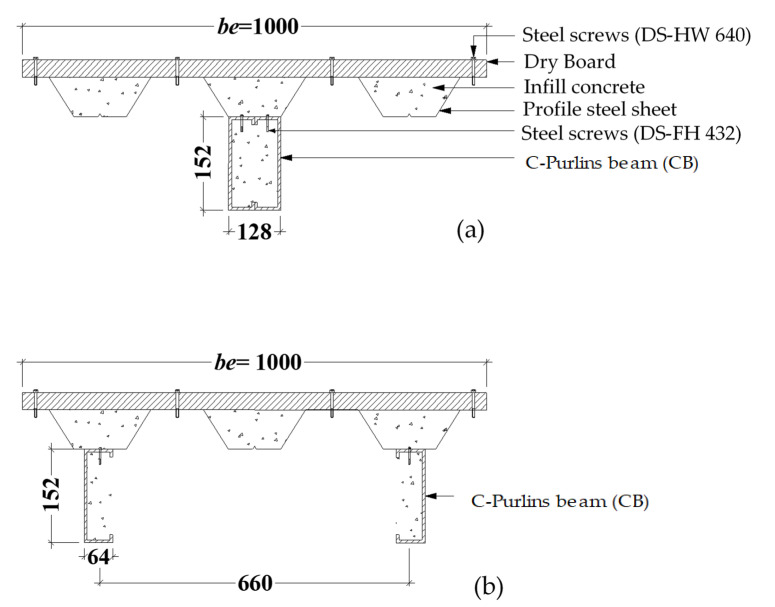
Cross-section of the CBPDS specimens (units in mm); (**a**) Double face-to-face (DF), and (**b**) Double Separate (DS).

In this study, six (6) full-scale beam–slab specimens were prepared using double steel C-Purlins beams connected from top to flange with profile steel sheet (PSS) deck slabs covered with dry board (DB) sheets. Each specimen was filled with a concrete mixture containing 0%, 50%, or 100% recycled aggregate. In addition, each specimen’s beam was fabricated with two pieces of C-Purlins either connected face-to-face or separated. The investigated CBPDS specimens’ nomenclature is described in Figure 1, and details are provided in Table 1. All sections (C-Purlins, PSS, and DB) of the CBPDS specimens were connected using self-tapping steel screws for quick and simple assembly. The typical details of the investigated CBPDS specimens are shown in Figure 2 and Figure 3.

**Figure 4:**
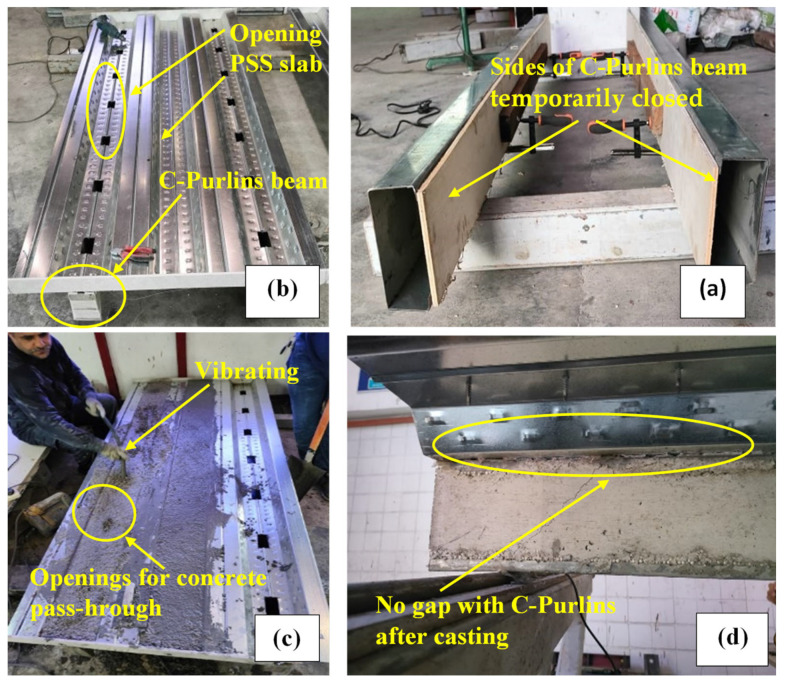
Pouring the concrete inside the CBPDS specimens; (**a**)cover the C-Purlins sides, (**b**) provide openings, (**c**) pouring the concrete with vibrating, and (**d**) No concrete gap.

This study adopted a new technique for filling CBPDS specimens with concrete that provided several equally distributed (40 × 80 mm at 340 mm c/c) openings along the contact surface between the deck slab and the beam, as shown in Figure 4. This technique was considered more practical and easier to accomplish at the construction site compared to the existing method of pouring concrete inside the CFST beams by placing them vertically until they set [21,47]. Generally, concrete located at these openings (interlock points) was expected to acting as a shear connector, to further improve the connection behavior between the slab (PDS) and beam (CB) parts of this composite CBPDS specimen, in addition to the self-tapping steel screws (DS-FH432) that provided extra length for nailing the concrete core of the CFST beams (see Figure 2 and Figure 3). During the concrete casting process, a vibrating device was used to better distribute infill material inside the specimen’s sections (see Figure 4). Following this, the PSS deck slab was covered with DB sheets that were fixed in place using self-tapping screws (DS-HW640).

### 2.2. Material Properties

**Figure 5:**
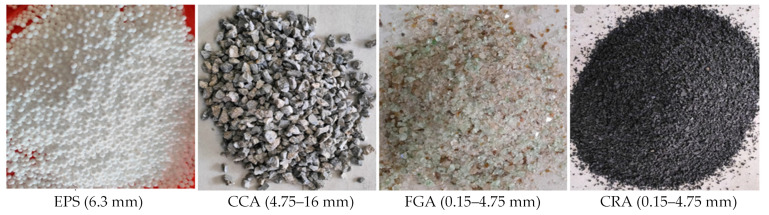
The types of recycled aggregates.

**Figure 6:**
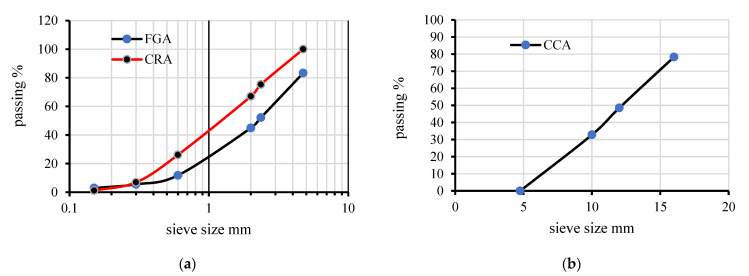
Sieve analysis for the recycled aggregates. (**a**) Fine aggregate FGA and CRA; (**b**) Coarse aggregate CCA.

Three different concrete mixtures were prepared, each containing a different amount of recycled aggregates (0%, MC0; 50%, MC50; and 100%, MC100) which were replaced by volume with raw fine and raw coarse aggregates. The concrete mixtures were mainly prepared using Malaysian Portland cement (MS EN 197-1 CEM II/B-L 32.5N), washed sand (density, 1570 kg/m^3^), and crushed limestone coarse aggregates (density, 1498 kg/m^3^). A high water/cement ratio (0.5) was used to increase the concrete mixture’s workability while it was poured inside the CBPDS specimens. In this study, combinations of four different waste materials were used as recycled aggregates (EPS, CCA, CRA, and FGA), as shown in Figure 5. Sieve analysis was performed in the laboratory for three recycled materials, as shown in Figure 6. The densities of these recycled aggregates were 1278 kg/m^3^ for the CCA, 9.5 kg/m^3^ for the EPS, 600 kg/m^3^ for the CRA, and 1220 kg/m^3^ for the FGA. Table 2 presents the proportions of these concrete mixtures. After 28 days, three cubes (100 mm) were taken from each mixture and tested in accordance with BS1881-Part116 1983. In general, at the preliminary lab work stage, five concrete mixtures that contain different combinations of recycled aggregates were suggested. Then, one mixture was chosen to apply as infill recycled concrete material for the investigated CBPDS specimen, specifically the mixture with optimal content of recycled aggregates and better concrete strength compared to normal concrete NC.

In addition, three steel coupons were prepared, using steel PSS and C-Purlins, and tested in accordance with ASTM E8/E8M-2009. This study used the same Primaflex DB sheet that was used in [7,9,10]. Table 3 presents the properties of these materials (C-Purlins, PSS, DB, and concrete mixtures). Lastly, two types of self-tapping steel screw were used to connect the parts of the CBPDS specimens, which are DS- and DS-FW640 [7,9,10].

### 2.3. Test Setup

**Figure 7:**
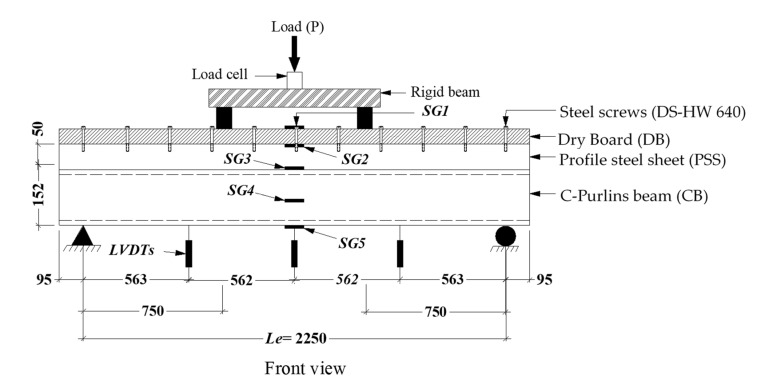
Typical test setup (all units in mm).

All CBPDS specimens were tested as a simply supported beam, using the test setup shown in Figure 7, all dimensions in mm. During the loading test, three LVDTs, positioned under the specimens, recorded their vertical deflection, and five strain gauges (SG), located at the mid-spans, measured changes in the longitudinal strain values of the C-Purlin beams, PSS slabs, and DB sheets. The load was applied at two points and was increased using a continuous increment (6–8 kN/min). The deflection, load, and strain values were collected and saved in a PC using a data logger device.

## 3. Discussion of Test Results

### 3.1. Typical Failure Mode

**Figure 8:**
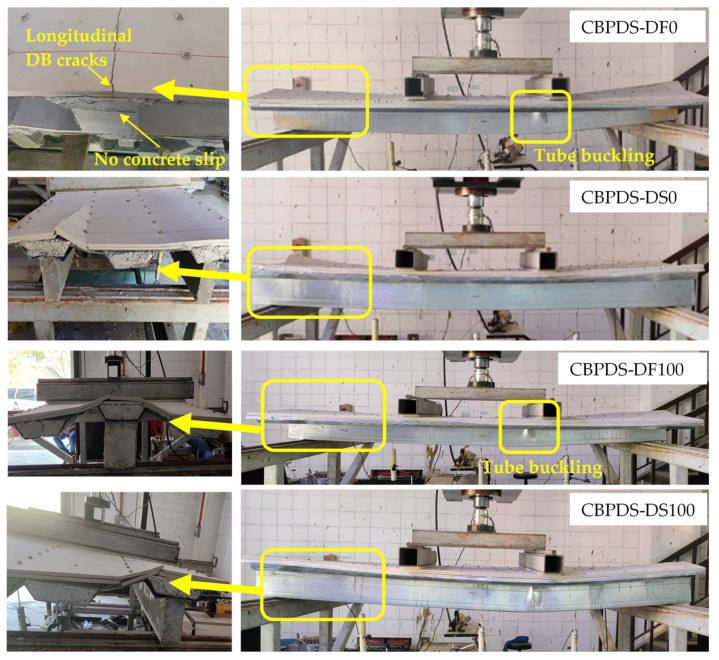
Bending behavior of the CBPDS double-filled and double separate specimens.

**Figure 9:**
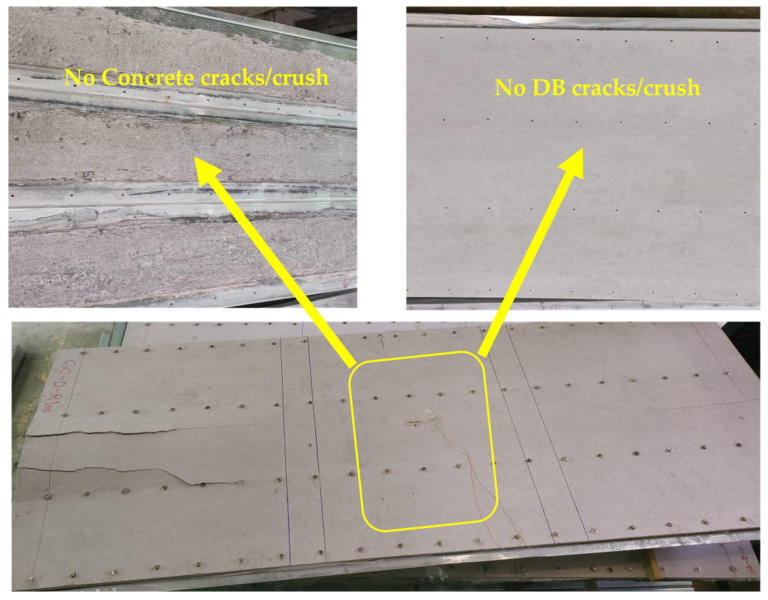
Bending behavior of the CBPDS in mid-span for all specimens.

The tested CBPDS specimens showed similar bending behavior with compressive stresses at the top flange of the slab part (PDS), and tension stresses at bottom flanges of the C-Purlins beam part (CB). Generally, this performance was observed for all specimens regardless of the recycled aggregate composition of their concrete cores (0%, 50%, and 100%), and for both C-Purlins beam configurations. The specimens with double C-Purlins filled with the highest percentages of recycled aggregates (specimens CBPDS-DF100 and CBPDS-DS100) showed a typical steel outward buckling failure similar to the corresponding control specimens CBPDS-DF0 and CBPDS-DS0 (0% recycled aggregates), as compared in Figure 8. Tube buckling occurred at the top flanges of C-Purlins when the loading test passing 85% of the specimens’ ultimate bending capacity. In addition, none of the DB sheets or infill concrete materials inside the PSS slab recorded any crushing failure, as shown in Figure 9. This was due to the influence of infill concrete materials inside the PDS part, even with high percentages of recycled aggregates (50% and 100%), which is considered an important contribution to this CBPDS system.

**Figure 10:**
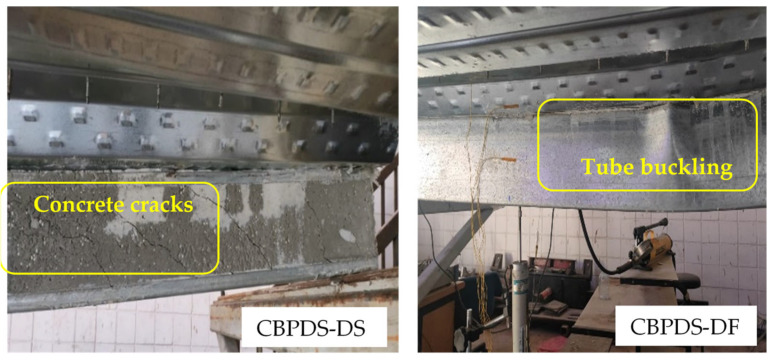
Buckling and concrete cracks for beam in CBPDS specimens.

**Figure 11:**
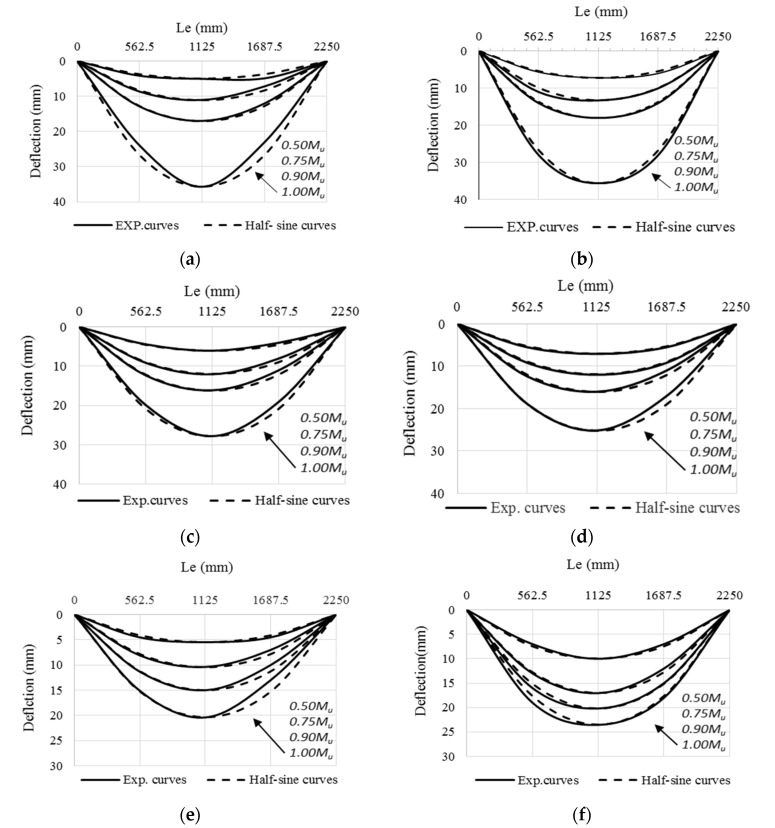
Deflection curves of CBPDS specimens. (**a**) CBPDS-DF0; (**b**) CBPDS-DS0; (**c**) CBPDS-DF50; (**d**) CBPDS-DS50; (**e**) CBPDS-DF100; (**f**) CBPDS-DS100.

In addition, the same failure mode was observed for specimens CBPDS-DS0, CBPDS-DS50, and CBPDS-DS100 (double separate beam). However, the concrete core of these beams experienced earlier cracking failure than those with face-to-face connections; the latter configuration achieved better concrete confinement behavior, as it was fabricated in a tube shape [21,45]. Generally, these cracks started when about 70–75% of the specimens’ ultimate bending capacity was achieved, and gradually increased with increases in the loading test, as shown for specimen CBPDS-DS100 in Figure 10. Furthermore, no concrete slipping failures from the C-Purlins or PSS sections were recorded for any of the tested specimens, even at the extreme bending stage. This perfect bonding behavior between the concrete core and the steel section of CBPDS specimens was achieved due to the openings provided to casting the concrete and the embedded length of self-tapping screws used to fix the PSS to C-the Purlins beams. This behavior was recorded for all specimens, even those containing 100% recycled aggregates with double separate C-Purlins beams. All specimens showed a typical deflection behavior similar to the half-sine curve (Figure 11).

### 3.2. Bending Behavior and Strength

**Figure 12:**
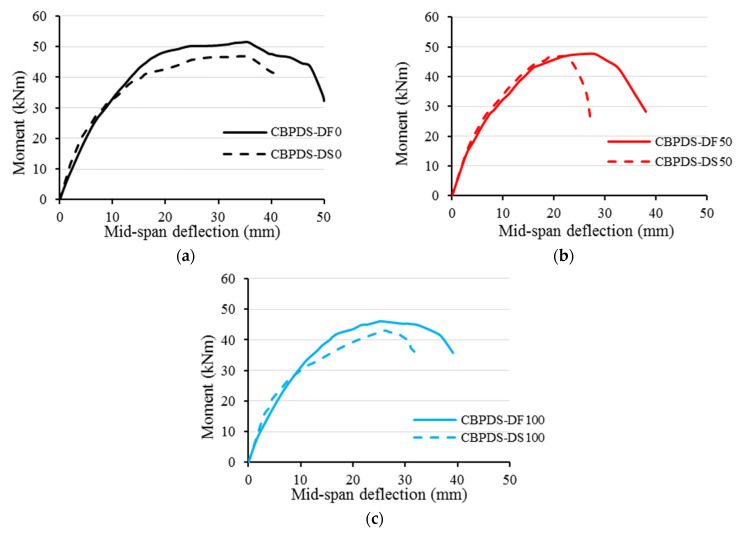
Moment–deflection relationships of specimens. (**a**) MC0; (**b**) MC50; (**c**) MC100.

Generally, CBPDS specimens’ bending behavior was expressed as a moment–deflection relationship, as shown in Figure 12. Regardless of the recycled aggregates content of infill concrete material (0%, 50%, or 100%) and the C-Purlins’ configuration (face-to-face or separate connections), all specimens showed elastic behavior at the early loading stage (at 50–60% of each specimen’s ultimate capacity). Next, these curves showed an elastic–plastic behavior with continuously dropping slopes until the peak points were achieved at the curve. After that, their moment–deflection curves rapidly decreased due to the increased steel buckling of the PSS slabs and C-Purlins beams as well as increasing concrete cracks inside the beams at the shear span distance. However, the specimens containing 50% and 100% recycled aggregates (CBPDS-DF50, CBPDS-DS50, CBPDS-DF100, and CBPDS-DS100) exhibited slightly lower bending behavior than their corresponding control specimens (CBPDS-DF0 and CBPDS-DS0), specifically at the plastic loading stage, due to the lower compressive strength of their concrete mixtures (MC50 and MC100).

**Figure 13:**
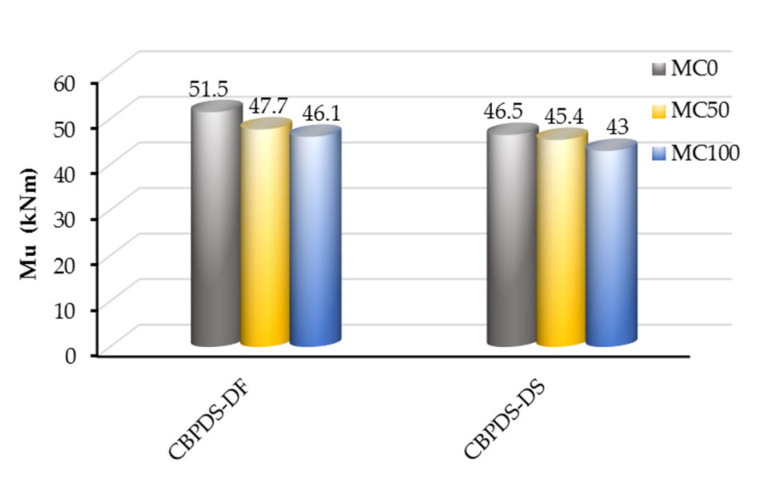
Ultimate moment capacity of specimens.

The ultimate bending strengths (M_u_) obtained at the peak values of all tested specimens’ moment–deflection curves are recorded in Table 1. In addition, Figure 13 compares the M_u_ values of the CBPDS specimens based on their infill concrete mixture (MC0, MC50, or MC100) and according to their C-Purlins beam configuration (CBPDS-DF and CBPDS-DS). Generally, regardless of the C-Purlins connection type, specimens’ M_u_ values decreased as the recycled aggregate content increased, which is logical, as the concrete’s compressive strength usually decreased accordingly, as shown earlier in Table 2. For example, the M_u_ value decreased approximately 7% when 50% of the raw aggregate was replaced with recycled aggregates, from 51.5 kN.m for specimen CBPDS-DF0 (control specimen) to 47.7 kN.m for specimen CBPDS-DF50. The M_u_ value decreased approximately 11% (46.1 kN.m) when 100% recycled aggregate was used (specimen CBPDS-DF100). Therefore, it can be concluding that replacing 100% of the raw aggregate with a combination of recycled aggregates reduced the suggested composite beam–slab specimens’ concrete cores’ self-weight by approximately 15%; their M_u_ values decreased approximately 9–11% with little effect on overall bending behavior.

### 3.3. Relationships of the Moment vs. Longitudinal Strain

**Figure 14:**
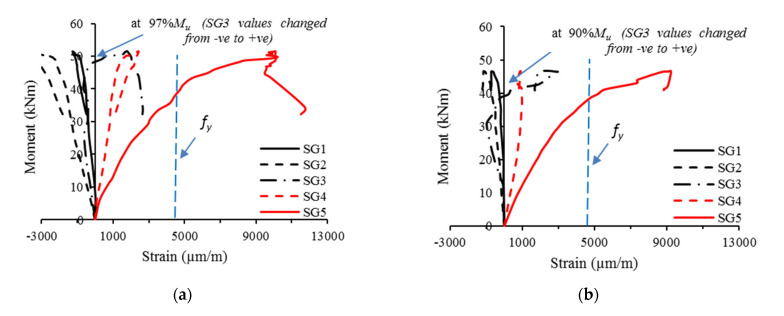

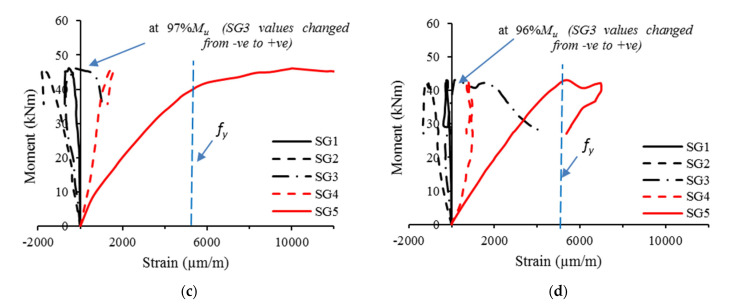
Moment versus strain relationships for specimens. (**a**) CBPDS-DF0; (**b**) CBPDS-DS0; (**c**) CBPDS-DF100; (**d**) CBPDS-DS100.

Figure 14 presents specimens’ moment–strain relationships, specifically for specimens with 0% and 100% recycled aggregates. In general, the SG1 and SG2 strain gauges measured negative strain values; this confirmed that the top fibers of specimens were subjected to compression stress due to the high bending stress at their mid-spans. In contrast, the SG5 strain gauge (fixed at the beams’ bottom flanges) showed continuous increases in positive strain values. The SG3, located at the connection surface between the C-Purlins and PSS deck slab, first measured marginally negative strain values, and then measured positive strain values when loads reached 90–97% of the specimens’ ultimate strength. This performance indicated that each CBPDS specimen’s neutral axis (NA) was within the top quarter of its beam’s cross-section; then, the NA moved upward until it was slightly above the beam–slab connection level and became slightly above the bottom flange of the PSS (see Figure 14). Moreover, the negative strain values of SG2 (fixed at PSS’ top flanges) did not reach the yielding limit even at the extreme loading stage indicating that the compression strength of specimens’ PSS slabs did not reach their yielding limits. In contrast, SG5 (fixed at the C-Purlin’s bottom flange) readings indicated that steel C-Purlins’ tensile strengths started to achieve their yielding limits when the bending moment reached approximately 85–90% of the specimens’ ultimate bending strength. Lastly, increasing the recycled aggregate content in the concrete to 100% had no effect on the moment–strain relationships of CBPDS specimens.

### 3.4. Ductility Index

**Figure 15:**
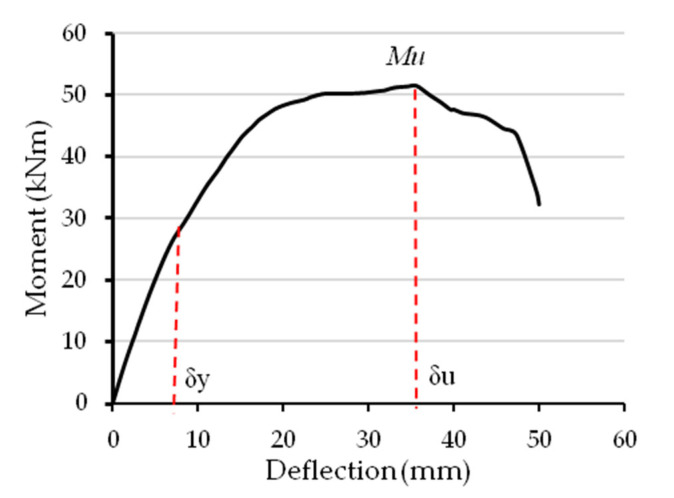
Typical moment–deflection curve for estimating the DI.

**Figure 16:**
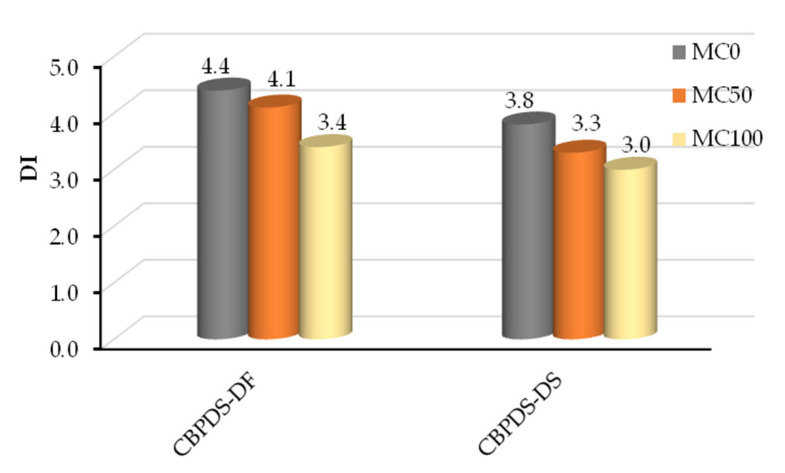
Ductility index (DI) of tested specimens.

Typically, for all tested specimens, the DI value was estimated from the deflection ratio at the ultimate bending limit (δ_u_) to that at the yielding limit (δ_y_) [48], as illustrated in Figure 15. Specimens’ DI indices are compared in Figure 16 with reference to their C-Purlins connection type (CBPDS-DF or CBPDS-DS). Generally, regardless of beam configuration, the highest DI value was achieved by control specimens with 0% recycled aggregate content; this value gradually decreased as recycled aggregate content increased. For example, control specimen CBPDS-DF0′s DI value was approximately 4.4, compared to DI values of 4.1 (−7.0%) and 3.4 (−22%) when specimens’ infill concrete materials contained 50% and 100% recycled aggregates, respectively. Generally, specimens with double separate C-Purlins beams (CBPDS-DS) showed similar performance to specimens with face-to-face connection beams (CBPDS-DF), but with slightly lower DI values, as the specimens with face-to-face beam configurations achieved better concrete confinement, which led to improved bending performance.

## 4. Theoretical Model Development

**Figure 17:**
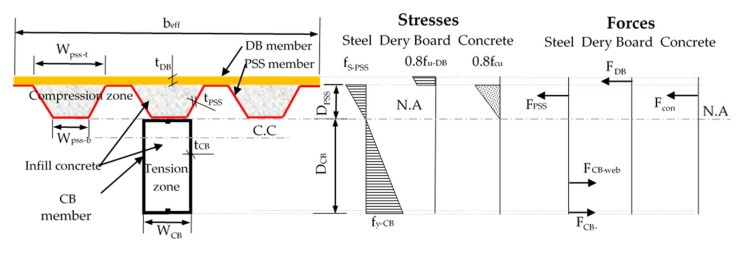
Stress block diagram of CBPDS system.

The steel tube members were classified as either slender, non-compact (semi-compact), or compact sections, according to how they buckle under compression stress [20,49]. Specifically, the AISC-2010 standard [50] classifies rectangular CFST members. The suggested CBPDS specimens’ beams were fabricated using double C-Purlins members filled with concrete materials, and classified as slender sections [20,21]. The PSS deck slabs in this composite specimen were also classified as slender sections, as the PSS sections were thin relative to the flat distance of their cross-sections, which was evident when the PSS’ top flanges buckled under compression stress (at mid-span distance due to their bending behavior). However, to date, there is no analytical model that can predict the nominal bending capacity (M_n_) of the investigated composite beam–slab member (CFST beam carrying a PSSDB/PDS slab). Therefore, in this study, a new theoretical model was developed using the stress block theory, as shown in Figure 17. The main assumptions adopted in the development of this model are as follows:

1. This model is limited to the concrete-filled composite CBPDS specimens prepared with rectangular steel tube beams and PSS deck slabs covered with dry board (DB) sheet subjected to static bending loads.
2. The concrete cores achieved full interaction with the steel tube beams and PSS deck slabs, as no slip failures were recorded for the tested specimens (see Section 3.1).
3. The steel tube beams achieved full interaction with the PSS slabs, as no horizontal/vertical separations were recorded for the tested specimens (see Section 3.1).
4. The effects of C-Purlins lips were ignored.
5. The effects of the infill concrete section below the NA was ignored, as it was subjected to tension stress and faced cracking failure [20].
6. As both the steel tube beam and PSS slab are slender sections, a pure elastic behavior has been assumed for these sections, which is limited to f_y-CB_ at the C-Purlins’ bottom flange (maximum tension stress) [20], as the tensile strain values shown in Figure 14 (SG5). Furthermore, the PSSs’ top flange (maximum compression stress) is limited to the buckling stress (f_s-pss_), as it has not reached the yield limit (see SG2 in Figure 14). Thus, the stress value of f_s-pss_ is estimated via liner interpolation with the value of f_y-CB_.
7. A lower compression stress equal to 0.8f_cu_ and 0.8f_u-DB_ is adopted for the concrete-filled PSS slab and DB sheet, respectively, as no crush failures were recorded for these two sections until the end of specimen tests (as previously discussed in Section 3.1 and shown in Figure 9).
8. To simplify this model, the position of NA (y_n_) is assumed to be located at the beam–slab connection level (connection level of C-Purlins with PSS), as previously discussed in Section 3.3, based on the readings of strain gauge SG3, as highlighted in Figure 14.
9. Lastly, for design purposes, reduction factors (Ø) equal to 0.8 and 0.7 are suggested for predicting the M_n_ values of CBPDS specimens with double face-to-face C-Purlins beam and double separate beams, respectively.

Based on the above assumptions and details presented in Figure 17, the forces over the CBPDS specimens’ sections have been estimated to form the final expression of the newly suggested theoretical model, summarized as follows:

M_n_ = Ø (F_CB-flange_. Y_CB-flange_ + F_CB-web_ . Y_CB-web_ + F_PSS_ . Y_PSS_ + F_con_ . Y_con_ + F_DB_ . Y_DB_) (1)

where the detail of forces is as follows,
F_CB-flange_= f_y-CB_ . A _CB-flange_
= f_y-CB_ . (t_CB_ . W_CB_)(only the bottom flange area)F_CB-web_= f_y-CB_ . A _CB-wed_
= f_y-CB_ . (2 . t_CB_ . D_CB_) . 0.5
F_PSS_= f_s-pss_. A _PSS_
= (f_y-CB_/D_CB_ .D_PSS_) . A _PSS_ (excluded the bottom flanges area)F_con_= 0.8f_cu_ . A _con_= 0.8f_cu_ . (3. (W_pss-t_ + W_pss-b_)/2 . D_PSS_) . 0.5F_DB_= 0.8f_u-DB_ . A _DB_
= 0.8f_u-DB_ . (b_eff_ .t_DB_)
Y_CB-flange_= the distance of F_CB-flange_ from the N.A = D_CB_ − 1/2.t_CB_
Y_CB-web_
= the distance of F_CB-web_ from the N.A = 2/3.D_CB_
Y_PSS_
= the distance of F_PSS_ from the N.A = 2/3.D_PSS_
Y_con_
= the distance of F_con_ from the N.A = 2/3.D_PSS_
Y_DB_
= the distance of F_DB_ from the N.A = D_PSS_ + 1/2.t_DB_Ø = 0.8 for CBPDS specimens (double face-to-face C-purlins beam) 
0.7 for CBPDS specimens (double separate C-purlins beam)

**Figure 18:**
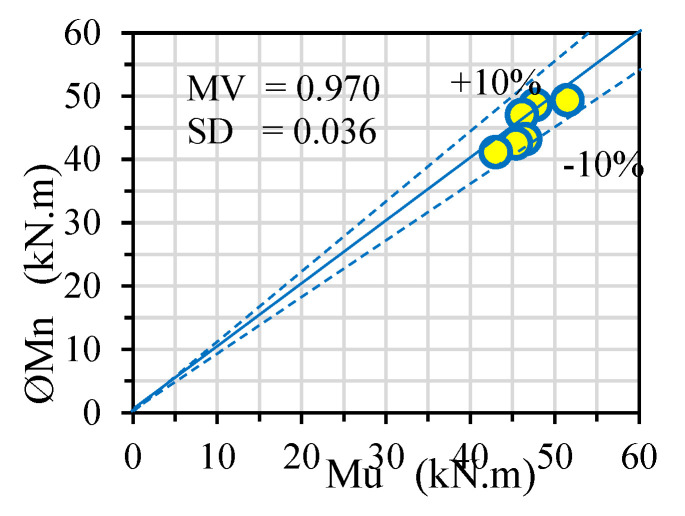
Compare the nominal bending (ØM_n_) values with the experimental results (M_u_).

The M_n_ values of the investigated CBPDS specimens are predicted using the new analytical model and compared with those obtained from the corresponding test results in Table 1 and Figure 18. A lower estimate was achieved using this developed model with a sufficient mean value and standard deviation of 0.970 and 3.6%, respectively.

## 5. Conclusions

The conclusions are summarized as follows:

➢ Filling CBPDS specimens with infill material containing 50% and 100% of recycled aggregate had little effect on their flexural behavior. Thus, using a combination of different recycled aggregates (EPS, CCA, CRA, and FGA) in the specimens’ concrete cores achieved a sufficient contribution, by reducing their self-weight approximately 15–18% and using less raw aggregate, which was considered an environmental improvement.
➢ Increasing the recycled aggregate content of the CBPDS specimens’ concrete materials to 50% and 100% reduced their ultimate bending strength by approximately 7–11% compared to the control specimen, due to reductions in their concrete compressive strengths. However, the specimen beams with double separate C-Purlins sections had slightly lower bending capacities than those with face-to-face connections. For example, the specimen with face-to-face C-Purlins beam and filled with 100% recycled concrete materials (CBPDS-DF100) achieved bending capacity equal to 46.1 kN.m, where this capacity reduced to 43 kN.m only when the C-Purlins beams were separated (CBPDS-DS100).
➢ Accordingly, the CBPDS specimens’ ductility indexes were reduced with increases of recycled aggregate content in their concrete cores; this is due to the reduction in their bending capacities. For example, the CBPDS-DF specimen filled with normal concrete (0% recycled aggregates) achieved a ductility index equal to 4.4, and this value reduced to 4.1 and 3.4 when the same specimen was filled with concrete mixture content 50% and 100%, respectively, of recycled aggregate.
➢ Based on the available experimental results, the newly developed theoretical model reasonably predicted the nominal bending capacity of the composite CBPDS with an acceptable mean value (0.970) and standard deviation (3.6%).
➢ Notably, further investigations are required for these composite CBPDS specimens concerning the influence of varied parameters and loading scenarios that have not yet been studied. The developed theoretical model needs further validation with more empirical and numerical data for improvement, in order to make it generally applicable to this type of composite beam–slab system (CFST beam carrying PDS slab system).

## Figures and Tables

**Table 1:** Specimens’ designation and tests results.

Specimens Designation	Double C-Purlins Configuration	Recycled Aggregates Contains	M_u_ (kN.m)	Load Ratio	ØM_n_ (kN.m)	ØM_n_/M_u_
CBPDS-DF0	face-to-face	0%	51.5		49.4	0.957
CBPDS-DF50	face-to-face	50%	47.7	0.93	48.5	1.017
CBPDS-DF100	face-to-face	100%	46.1	0.89	46.9	1.018
CBPDS-DS0	separate	0%	46.5		43.1	0.927
CBPDS-DS50	separate	50%	45.4	0.97	42.4	0.937
CBPDS-DS100	separate	100%	43.0	0.92	41.0	0.955
			Mean Value			0.970
			Standard Deviation			0.036

**Table 2:** Proportion of the concrete mixtures (kg/m^3^).

Concrete Mixture ID	Cement	Fine Aggregate	Coarse Aggregate	Water	EPS (%)	CCA (%)	CRA (%)	FGA (%)	Density
MC0	395	700	1115	200	-	-	-	-	2247
MC50	375	560	781	200	15	15	10	10	2086
MC100	375	420	446	200	30	30	20	20	1950

**Table 3:** Physical properties of the tested materials.

Materials	Yield Strength (MPa)	Ultimate Strength (MPa)	Elastic Modulus (GPa)
C-Purlin	492.0	536.0	210.0
Profiled Steel Sheet (Peva 50)	434.0	464.0	213.0
Dry board (Primaflex)		22.0	8.0
MC0 (0% recycled aggregates)		20.1	21.0
MC50 (50% recycled aggregates)		17.2	19.5
MC100 (100% recycled aggregates)		11.2	15.7

## Data Availability

Data are presented in the article.

## Abbreviations

Profile steel sheet (PSS), Cold-formed beam (CB), Dry board (DB), PSS slab covered with DB (PSSDB/PDS), center of specimen’s cross-section (C.C), depth of C-Purlins (D_CB_), depth of PSS (D_PSS_), thickness of DB (t_DB_), modulus of elasticity for concrete (E_c_), modulus of elasticity for steel (E_s_), concrete cube compressive strength at 28 days (f_cu_), ultimate tensile strength of steel (f_u_), yield tensile strength of steel (f_y_), force on the C-Purlins beam’s flange (F_CB-flange_), force on the C-Purlins beam’s web (F_CB-web_), force on the concrete part inside the PSS (F_con_), force on the DB part (F_DB_), force on the PSS part(F_PSS_), ultimate bending moment (bending strength capacity; M_u_), effective length (L_e_), steel C-section thickness (t), strain gauge (SG), area of the PSS cross-section (A _PSS_),W_pss-t_ & W_pss-b_ are the top and bottom ribs’ width of the PSS.
